# Precursor Lesions for Sporadic Pancreatic Cancer: PanIN, IPMN, and MCN

**DOI:** 10.1155/2014/474905

**Published:** 2014-03-24

**Authors:** M. Distler, D. Aust, J. Weitz, C. Pilarsky, Robert Grützmann

**Affiliations:** ^1^Department of Visceral, Thoracic, and Vascular Surgery, University Hospital Carl Gustav Carus Dresden, TU Dresden, Fetscher Street 74, 01307 Dresden, Germany; ^2^Institute for Pathology, University Hospital Carl Gustav Carus Dresden, TU Dresden, Fetscher Street 74, 01307 Dresden, Germany

## Abstract

Pancreatic cancer is still a dismal disease. The high mortality rate is mainly caused by the lack of highly sensitive and specific diagnostic tools, and most of the patients are diagnosed in an advanced and incurable stage. Knowledge about precursor lesions for pancreatic cancer has grown significantly over the last decade, and nowadays we know that mainly three lesions (PanIN, and IPMN, MCN) are responsible for the development of pancreatic cancer. The early detection of these lesions is still challenging but provides the chance to cure patients before they might get an invasive pancreatic carcinoma. This paper focuses on PanIN, IPMN, and MCN lesions and reviews the current level of knowledge and clinical measures.

## 1. Introduction

Pancreatic cancer is the fourth leading cause of cancer death in the USA [1]. The high mortality rate is mainly caused by the lack of highly sensitive and specific tools to detect the disease in an early stage, and therefore most of the patients are diagnosed in advanced tumor stages. Currently, surgical resection is the only curative treatment option. However, only a small number of patients (30–40%) present with a resectable tumor at the time of diagnosis. The overall five-year survival after pancreatic head resection for cancer has been reported to range between 10 and 25% [2, 3]. The five-year survival rate can be significantly improved for patients with pancreatic cancer when surgery is possible and patients additionally receive adjuvant therapy [4, 5]. However, there are patients that relapse shortly after the surgery and, therefore, have only a limited life span even after complete resection of the tumor.

**Figure 1:**
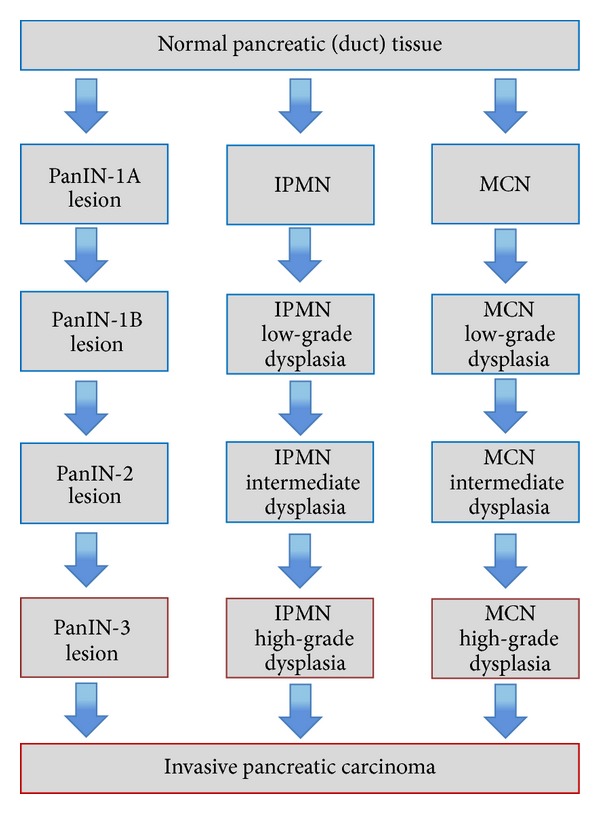
Model of three distinct morphological pathways to invasive pancreatic carcinoma.

Current knowledge about pancreatic carcinogenesis postulates—analogous to other carcinomas—a stepwise progression from intraepithelial neoplasia to invasive cancer [6, 7]. Studies of resected pancreata from patients with a family history of pancreatic cancer or from patients with ductal adenocarcinoma of the pancreas (PDAC) demonstrated that many patients have multifocal, microscopic pancreatic intraepithelial neoplasms (PanINs) surrounding the tumor and in the remainder of the pancreas [8–11]. Furthermore, cystic lesions of the pancreas like intraductal papillary mucinous neoplasms (IPMNs) or mucinous cystic neoplasms (MCNs) are well known as precursor lesions for pancreatic cancer (Figure 1) [12–15]. Early detection and treatment of the abovementioned precursor lesions could probably save patients from advancing to invasive pancreatic cancer. In this review, a summary of the most important precursor lesions for pancreatic cancer, that is, PanIN, IPMN, and MCN, is given.

## 2. Pancreatic Intraepithelial Neoplasia (PanIN)

**Figure 2:**
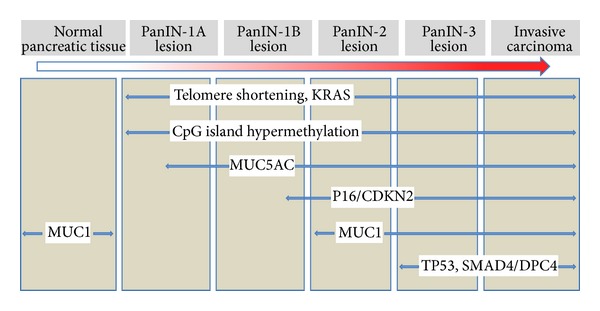
Compendium of molecular changes during the PanIN-progression model. Adapted from [6, 18].

The most important and well-known precursor of a PDAC is pancreatic intraepithelial neoplasia (PanIN). For several decades, this noninvasive ductal lesion was described using multiple terminologies. Hruban et al. first presented the nowadays generally accepted “PanIN scheme” to classify these lesions in 2001 (Figure 2) [16, 17].

### 2.1. Histology

**Figure 3:**
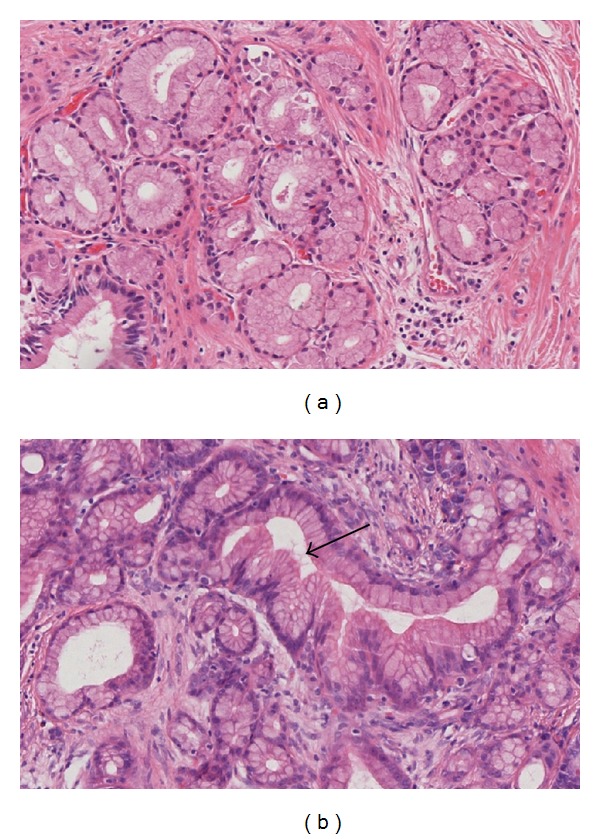
(a) PanIN 1A lesion with flat epithelium, basal nuclei, and abundant supranuclear mucin. (b) PanIN 1A and 1B (arrow) lesion with papillary architecture and slight nuclear atypia (H&E ×20).

**Figure 4:**
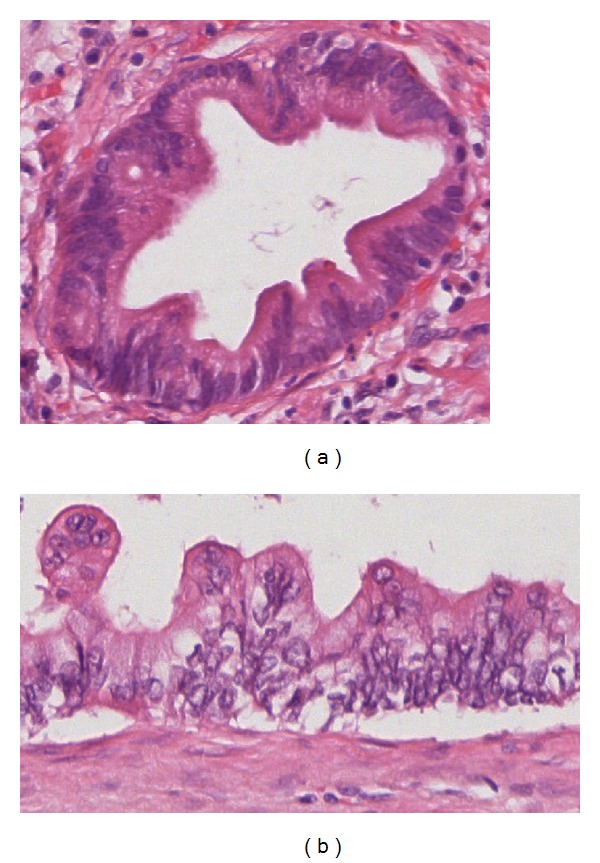
(a) PanIN-2 lesion with moderate cytological atypia, retained nuclear polarity, and partially papillary architecture (H&E, 20x); (b) PanIN-3 lesion with severe cytological atypia, lost nuclear polarity, and papillary architecture (H&E, 40x).

A PanIN is a microscopic (usually <5 mm) flat or papillary lesion arising in the small intralobular pancreatic ducts [17]. These lesions are characteristically asymptomatic. PanINs are composed of columnar to cuboidal cells with varying amounts of mucin and varying degrees of cytological and architectural atypia [15]. They are classified into three grades:* PanIN-1A* (flat) and* PanIN-1B* (papillary) are low-grade lesions with minimal cytological and architectural atypia (Figure 3).* PanIN-2* lesions (intermediate-grade PanIN) show mild to moderate cytological and architectural atypia (e.g., nuclear pleomorphism, nuclear crowding, and nuclear hyperchromasia) with frequent papillae (Figure 4) [6, 15]. High-grade PanINs (*PanIN-3*) are characterized by severe cytological and architectural atypia.* PanIN-3*  is also referred to as* “carcinoma in situ.” *All PanINs are noninvasive lesions that do not trespass the basement membrane [16, 17].* PanIN-3* lesions usually have a papillary morphology but can also present with a flat or cribriform pattern (Figure 4). In addition, there are some rare variants of PanINs (e.g., intestinal type, foam gland type, and oncocytic type) that do not seem to have any specific clinical significance [15].

Formal pancreatic carcinogenesis is thought to progress from low-grade to high-grade PanIN and then to invasive cancer; this histological progression is paralleled by the accumulation of genetic changes (Figure 2).

### 2.2. Immunohistochemical Characteristics

The immunohistochemical characteristics of PanINs vary with the grade of dysplasia. In particular, the apomucins MUC1, MUC2, and MUC5AC are frequently overexpressed in epithelial cancers of the gastrointestinal tract [14, 19]. MUC1 is responsible for the surveillance of lumen formation and is typically expressed by the pancreatic ducts and centroacinar cells. Furthermore, MUC1 is almost exclusively expressed in the higher-graded lesions (*PanIN-2/PanIN-3*) and often linked with an invasive PDAC [18, 20]. The intestinal mucin marker MUC2 and the intestinal differentiation marker CDX-2 are negative in common-type PanIN and PDAC. However, MUC2 and CDX-2 expression is seen in IPMNs with intestinal differentiation and therefore can be used for differentiation between PanINs and intestinal IPMNs [14, 19, 21]. In contrast to MUC1, MUC5AC is not expressed by the normal pancreatic ducts but, similar to MUC1, in the majority of invasive ductal carcinomas and moreover already in the early PanIN lesions [6, 18, 20, 22, 23]. Furthermore, the membrane-associated mucins MUC3 and MUC4 show a progressive increase in PanIN lesions of increasing dysplasia and are also highly expressed in ductal adenocarcinoma [20, 24, 25].

### 2.3. Genetic and Epigenetic Changes

As mentioned above, PanINs are part of the multistep tumor progression model in pancreatic cancer. Early genetic alterations include telomerase shortening, V-Ki-ras2 Kirsten rat sarcoma viral oncogene homolog (KRAS) activation, and inactivation of tumor suppressor genes cyclin-dependent kinase inhibitor p16 (p16)/cyclin-dependent kinase inhibitor 2A (CDKN2A) [17]. KRAS mutations are one of the earliest genetic abnormalities in the progression model for pancreatic cancer [26]. The most common activating point mutations are located in codons 12 and 13 of exon 2 of the KRAS gene (in over 90% of PDACs) [6, 27, 28]. Early KRAS mutation is thought to be the driver for the formation of pancreatic cancer [29].

Telomere shortening is another early event in the progression model of pancreatic cancer. Telomeres are structures at the end of the chromosomes with a protective effect. They prevent fusion between the ends of the chromosomes. Telomeres shorten with the age of the cell and the number of cell divisions. Shortened telomeres lead to an abnormal fusion of chromosomes with chromosome instability, promoting neoplastic progression in the cells [6, 30].

In addition to activating KRAS mutations, leading to increased proliferation, and telomere shortening, leading to chromosomal instability, the inactivation of three tumor suppressor genes (p16/CDKN2A, tumor protein 53 (TP53), and SMAD family member 4 (SMAD4/DPC4)) is relevant for the formation of pancreatic cancer. While the inactivation of p16/CDKN2A is already detectable in the early PanIN stages, the inactivation of TP53 and SMAD4/DPC4 is associated with later alterations in the tumor progression model [17]. The p16/CDKN2A gene is located on the short arm of chromosome 9, and almost all pancreatic carcinomas present a loss of the p16/CDKN2A function [31]. Inactivation of p16 leads to an inadequate progression through the G1 phase of the cell cycle.

The TP53 tumor suppressor gene encodes for the p53 protein that is involved in the regulation of the cell cycle, maintenance of the G2/M arrest, and the induction of apoptosis. Loss of p53 causes deregulation in cell death and cell division [18, 32].

The gene DPC4 (located on chromosome 18q) encodes for the SMAD4 protein that plays an important role in signaling through the transforming growth factor type *β* (TGF-*β*) pathway. SMAD4 has a growth-controlling effect by regulating the expression of specific genes [33, 34]. Therefore, loss of SMAD4 leads to a decreased growth inhibition and thereby supports the growth of cancer cells.

Epigenetic changes predominantly occur through methylation of CpG islands, which are located in the promoter regions of genes leading to gene silencing [35]. Numerous studies have demonstrated hypermethylation of several genes in patients with pancreatic cancer [36, 37]. A microarray analysis by Sato et al. showed that aberrant CpG island hypermethylation begins in early stages of PanINs, and its prevalence progressively increases during neoplastic progression [38]. Furthermore, the detection of aberrantly methylated genes by methylation-specific PCR in the pancreatic juice might be an interesting diagnostic tool for the future [39].

### 2.4. Clinical Relevance of PanIN

As mentioned above, PanIN lesions are precursor lesions in the stepwise progression from intraepithelial to invasive pancreatic neoplasia. This morphological progression is paralleled by an accumulation of genetic changes in which activating KRAS mutations are thought to be the driving force (Figure 2). Early detection of PanINs would present an opportunity to cure patients before they develop invasive pancreatic cancer, but unfortunately PanINs are not yet detectable by cross-sectional imaging or endoscopic ultrasound (EUS). Molecular markers in the pancreatic juice may help to solve this dilemma. The most appropriate approach to the pancreatic resection margin when it harbors a PanIN lesion (detected by intraoperative frozen section) is, however, not clear. In particular, no consensus has been achieved on how to handle* PanIN-3* lesions in the resection margin [40]. We would recommend an additional resection in cases with* PanIN-3 *lesions (in the resection margin), whereas an additional resection in* PanIN-1* and* -2 *lesions might not be necessary.

## 3. Intraductal Papillary Mucinous Neoplasm (IPMN)

IPMNs belong to the heterogeneous group of cystic pancreatic lesions with increasing incidence in recent years [41–43]. These cystic lesions were initially reported in the 1990s, and the term IPMN was established in the 2000 classification of the WHO [44]. IPMNs are tumors of the duct epithelium. Papillary epithelial proliferation and mucin production lead to cystic dilatation of involved ducts [43]. IPMNs have been proven to be invasive carcinoma precursors, and thus progression models from noninvasive intraductal tumors via borderline lesions to invasive carcinoma have been developed (Figure 1) [45–47].

### 3.1. Histology and Immunohistochemistry

Morphologically, IPMNs are subdivided into the main (MD-IPMN) and branch duct types (BD-IPMN) according to their site of origin. When the main and branch ducts are both involved, the terms “mixed duct type” and “combined duct type” are used [51, 52]. Due to the papillary epithelial proliferation and mucin production, MD-IPMNs typically show a dilatation of the main pancreatic duct >6 mm. BD-IPMNs show no dilatation of the main pancreatic duct but a communication of the cystic lesion with the main duct, typically well definable by MRI/MRCP imaging. Some IPMNs might be multifocal and therefore even after partial pancreatic resection there is a risk of progression of a synchronous cystic lesion [53, 54]. Depending on their degree of dysplasia and the presence or absence of an associated invasive carcinoma, IPMNs are subclassified by the WHO into low-grade dysplasia, intermediate-grade dysplasia, high-grade dysplasia, and IPMN with associated invasive carcinoma [55]. Approximately, one-third of patients with IPMN are associated with invasive carcinoma [12, 14, 51, 56]. On the basis of their histological and immunohistochemical characteristics, intestinal, pancreatobiliary, oncocytic, and gastric subtypes of IPMN are distinguishable [14, 57]. Differential diagnosis of histopathological subtypes is accomplished by histomorphological and immunohistochemical analysis of MUC expression (MUC1, MUC2, MUC5AC, and MUC6) and the intestinal marker CDX-2 (Table 1) [19, 57, 58].

**Figure 5:**
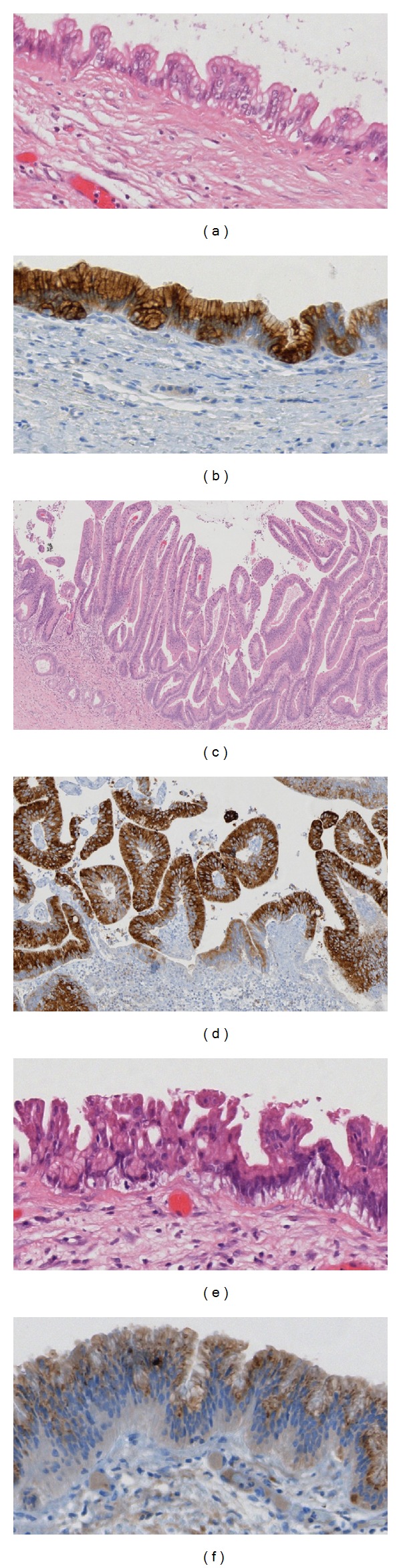
(a) and (b) IPMN gastric foveolar (branch duct) subtype with low-grade dysplasia, partially flat, partially papillary architecture, and basally oriented nuclei ((a); H&E 20x, (b); MUC5AC 20x); (c) and (d) IPMN intestinal subtype with low/intermediate dysplasia, long, finger-like papillae, and elongated nuclei ((c); H&E 4x, (b); MUC2 10x); (e) and (f) IPMN pancreatobiliary subtype with high-grade dysplasia, lost nuclear orientation, and complex papillae formation ((e); H&E 20x, (f); MUC1 20x).

Intestinal-type IPMN is characterized by tall columnar cells with elongated nuclei and amphophilic cytoplasm (similar to villous adenoma of the colorectum). The lesions frequently exhibit moderate or severe dysplasia [15]. These villous papillary neoplasms typically show an expression of MUC2, MUC5AC, and CDX-2 (Figure 5) (Table 1). A recent article by Kitazono et al. also demonstrated MUC4 expression in IPMN and especially in intestinal-type IPMN [59].

In contrast, the pancreatobiliary subtype of IPMN is characterized by branched papillae with high-grade intraepithelial neoplasms. The pancreatobiliary subtype is usually associated with an invasive component/IPMN-associated carcinoma. Immunohistochemical staining shows expression of MUC1 and MUC5AC (Figure 5) (Table 1) [60]. The third and rare subtype of MD-IPMN is the oncocytic type. This subtype mainly presents with oncocytic cells with a complex branched papillary structure and an eosinophilic cytoplasm (with intracytoplasmic lumina) mixed with goblet cells and mucin-containing cells [15]. Most of these cells show high-grade cellular atypia and carcinoma* in situ* modifications and convert into the infrequent oncocytic carcinoma when they become invasive. The oncocytic subtype is immunohistochemically characterized by a diffuse positivity for MUC5AC, MUC6, and a focal positivity for MUC1 or MUC 2 (Table 1). The gastric subtype, mainly found in BD-IPMN, is characterized by a foveolar glandular epithelium (similar to gastric foveolar cells) with formation of papillae. The cells present with oval-shaped nuclei with mild atypia and a slightly eosinophilic cytoplasm. Mitoses are rare, and most of the gastric-type lesions are low-grade lesions. Gastric-type IPMNs express MUC5AC and sometimes MUC6 (Table 1) [48, 49, 61].

While intestinal, pancreatobiliary, and oncocytic subtypes primarily originate in the main duct, the gastric subtype is typically derived from branch ducts. When IPMNs become invasive, two distinct types of invasive carcinomas typically develop, that is, tubular and colloid (mucinous noncystic) carcinomas (Table 1) [14, 45, 62]. While colloid carcinomas (characterized by abundant extracellular pools of mucin with floating neoplastic epithelium) usually arise from intestinal-type IPMNs, thetubular adenocarcinoma mainly arising from pancreatobiliary-type IPMN (invasive IPMN) and PDAC are morphologically similar, but they may not be the same. For instance, tubular-type invasive IPMN may harbor GNAS mutations. The distinction of these two tumor types has prognostic relevance because patients with colloid carcinomas have a better five-year survival rate than patients with tubular carcinomas [14, 48, 63, 64]. Other factors determining the prognosis of patients with IPMN are the presence of an invasive carcinoma, the lymph node ratio, and the histopathological subtype [56, 65, 66].

### 3.2. Molecular Pathology and Genetic Changes

Studies have identified a wide variety of genetic changes in IPMNs and some of them are similar to the findings in PDAC including KRAS, p16/CDKN2A, SMAD4, and TP53 genes [55, 67]. Other mutations, such as phosphatidylinositol-4,5-bisphosphate 3-kinase (PIK3CA) and v-Raf murine sarcoma viral oncogene homolog B1 gene (BRAF) mutations, are found in a small fraction of IPMNs [68, 69]. Recent studies by Wu et al. and Furukawa et al. added important information about the molecular anomalies of IPMN [70, 71]. They found that >96% of IPMNs have either a GNAS complex locus (GNAS) or a KRAS mutation and more than half of them have both mutations. The results of these studies indicate that GNAS mutations are common and specific for IPMN, and activation of G-protein signaling appears to play a pivotal role in IPMN. Although these mutations were found in all IPMN subtypes, GNAS mutations were more prevalent in the intestinal subtype, whereas KRAS mutations were more prevalent in the pancreatobiliary subtype [70–72]. Only a little is known about the characteristics of the oncocytic subtype of IPMN. KRAS mutations and TP53 overexpression are less frequently identified in oncocytic-type IPMN than in pancreatobiliary-type IPMN (17% and 11% versus 58% and 58%, resp.). However, the less frequent TP53 overexpression in the oncocytic subtype, associated with significantly lower rates of invasion and nodal involvement, correlates with a better outcome compared to pancreatobiliary-type IPMN [14, 73].

Current studies have evaluated the identification of molecular markers in the pancreatic juice. Siddiqui et al. demonstrated that the presence of GNAS in combination with* KRAS mutations* in pancreatic cystic fluid obtained by EUS-FNA improves the sensitivity for diagnosis of IPMN (accuracy 80%) compared to CEA or KRAS alone [74]. Another study by Kanda et al. identified GNAS mutations in 64.1% of the patients in a screening population with IPMN [75]. The same group detected TP53 mutations in the pancreatic juice in 9.1% of intermediate-grade IPMNs, 17.8% of PanIN-2 lesions, 38.1% of high-grade IPMNs, 47.6% of PanIN-3 lesions, and 75% of invasive pancreatic adenocarcinomas. Interestingly, no TP53 mutations were found in PanIN-1 lesions or low-grade IPMNs [76]. Although GNAS, KRAS, and TP53 mutations are helpful in identifying patients with IPMN, they occur early in the development of IPMNs and cannot be used to identify those individuals with high-grade dysplasia or invasive disease. Das et al. recently published seminal results regarding the detection of high-risk IPMN lesions. By analyzing the cyst fluid of IPMNs, they found that the monoclonal antibody Das-1 has 89% sensitivity and 100% specificity to detect high-risk/malignant IPMNs [77].

Moreover, recent reports demonstrated a level of hypermethylation in IPMN (e.g., p16/CDKN2A, cyclin-dependent kinase inhibitor 1C (CDKN1C), SRY- (sex determining region Y-) box 17 (SOX17)). An increasing number of hypermethylated loci are associated with an increasing grade of dysplasia [78].

### 3.3. Clinical Relevance of IPMN

In summary, IPMNs are premalignant cystic lesions usually arising from intestinal type IPMN, the tubular adenocarcinoma can be detected and treated before the onset of malignancy. A current challenge is the differentiation of cystic lesions (serous cystic neoplasm (SCN), mucinous cystic neoplasm (MCN), solid pseudopapillary neoplasm (SPN), and pseudocysts) and especially IPMN preoperatively. These cystic lesions appear different in clinical and radiologic diagnostic [47].  Table 2 summarizes the core characteristics of the different cystic pancreatic lesions. In particular, diagnostic classification of the IPMN subtypes (e.g., by cystic fluid) is important for individualized patient treatment.

Generally, resection of all MD-IPMNs (and mixed duct-type IPMN) and of BD-IPMNs with “worrisome features” is indicated. The recently published revised international consensus guidelines discuss this topic in detail [50].

Better markers are required to accurately identify patients who would benefit from surgical resection or could be placed under surveillance. The examination of pancreatic juice/cystic fluid seems to be a promising diagnostic option. Recently, Hara et al. showed that pancreatic juice cytology with MUC stain is highly reliable for the identification of the histological subtypes of IPMN [79]. Other authors reported about the identification of high-risk/malignant lesions by examination of the cyst fluid of IPMNs either by analyzing the MUC expression and cyst fluid cytokine levels (e.g., interleukin-1 beta) or by recognition of atypical cell components [80–83].

## 4. Mucinous Cystic Neoplasm (MCN)

MCNs of the pancreas are the most infrequent precursor lesions of pancreatic cancer. The accurate prevalence of MCN is difficult to assess; a recent publication by Valsangkar et al. reported about 23% of MCN in patients with resected cystic tumors of the pancreas [84]. MCNs mainly occur in women and are typically located in the pancreatic body and tail [42, 60, 85, 86]. These cystic lesions are almost solitary and the vast majority of MCNs are asymptomatic and found incidentally [15]. On imaging, the cysts appear septated and may contain calcifications. Like IPMNs, MCNs as mucinous pancreatic lesions have a high CEA and mucinous cytology in the cyst fluid [87, 88]. In contrast to IPMNs, MCNs usually present no obvious connection to the main pancreatic duct and no dilatation of the main pancreatic duct is typically detectable [12]. The prognosis for patients with noninvasive MCN is very favorable (five-year survival almost 100%), and also for patients who undergo resection for an invasive MCN the five-year survival rate is nearly up to 60% [86].

### 4.1. Histology and Pathogenesis

**Figure 6:**
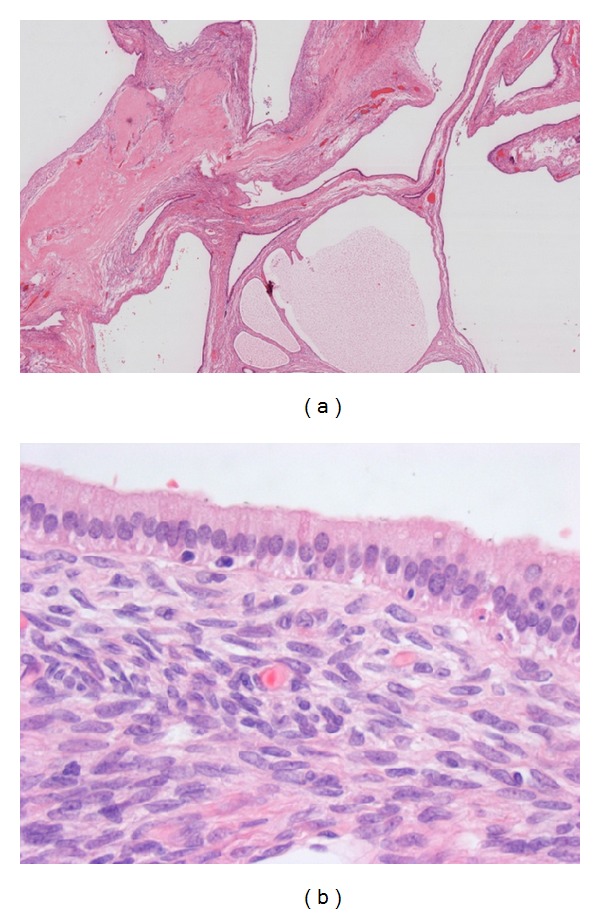
(a) Overview of a multicystic MCN (H&E 2x) with cuboidal to columnar epithelial lining, mild dysplasia, and underlying ovarian type stroma. (b) High power view of MCN with columnar epithelial lining and underlying ovarian type stroma (H&E 40x).

MCNs are cystic lesions that can grow very large. They present with a plain surface and a fibrous pseudocapsule with variable thickness and often with calcifications. The presence of mural nodules on the inside of the capsule correlates with malignancy [15, 89]. Microscopically, the epithelial lining of an MCN consists of columnar cells with a varying degree of dysplasia (Figure 6). On the basis of the degree of dysplasia (atypia), noninvasive MCNs are classified into low-grade dysplasia, moderate dysplasia, and high-grade dysplasia. About one-third of MCNs become invasive, and when they do, they usually form ductal adenocarcinomas [90].

Furthermore, one of the diagnostic clues of MCNs is the presence of an ovarian-like stroma underlying the neoplastic epithelium. The stroma expresses progesterone and estrogen receptors and can even undergo luteinization akin to the actual ovarian stroma [12, 89]. The epithelial cells of MCNs show immunoreactivity for epithelial markers including EMA, CEA, and cytokeratins 7, 8/18, and 19 and for the mucin markers MUC5AC and MUC2. MUC1 is usually expressed in high-grade lesions and invasive carcinomas. The ovarian-like stroma is positive for vimentin and smooth-muscle actin in addition to the progesterone receptor and estrogen receptor [15, 89].

### 4.2. Molecular Pathology

The molecular changes underlying MCN formation and progression are not entirely clear. KRAS mutations have been detected in MCNs with low-grade dysplasia and with increased frequency in advanced cases, while mutations of TP53, p16, and SMAD4/DPC4 have been mainly observed in high-grade dysplasia and invasive carcinomas [12, 23, 91, 92]. A recent exome sequencing analysis by Wu et al. showed that MCNs contain an average of 16 ± 7.6 somatic mutations (cf. with 27 ± 12 for IPMNs) and are characterized by KRAS, TP53, and RING-type zinc finger protein 43 (RNF43) mutations [93]. Interestingly, in this analysis, MCN harbored no mutations of GNAS and therefore GNAS may be a useful marker for the differentiation between IPMN and MCN.

### 4.3. Clinical Relevance of MCN

Although MCNs harbor a low risk of malignancy (prevalence of invasive carcinoma about 13%), surgery is indicated for any patient. Because, in particular, MCNs mainly occur in young patients and are mostly located in the body and tail of the pancreas, resection is the most appropriate and reliable treatment [42, 50].

Only frail and elderly patients for whom surgery is a higher risk should be placed under surveillance [15, 50].

## 5. Conclusion

There is still a lot to be learned about the biology of the three different precursor lesions of pancreatic cancer (PanIN, IPMN, and MCN). Early detection of these lesions would create an opportunity to prevent patients from developing invasive pancreatic cancer. However, clinical detection of these lesions is still challenging, even with modern high-resolution imaging methods. Currently,* PanIN* lesions (especially <5 mm) are not detectable using available imaging methods. Moreover, diagnostic differentiation of the different cystic pancreatic lesions (i.e., IPMN from MCN or SCN and SPN) is often complicated.

From this comes the need for reliable biomarkers to detect and differentiate precursor lesions for pancreatic cancer. Analyzing genetic alterations in pancreatic juice/cystic fluid may be a diagnostic option for the future, as preliminary studies have already demonstrated. Prospective studies to validate these markers are needed to put them into clinical practice.

## Figures and Tables

**Table 1:** Subtype classification of IPMN by immunohistochemical analysis and arising invasive carcinoma [48–50].

IPMN (subtype)	Expression profile	Invasive carcinoma
**Intestinal ** (MD-IPMN)	**MUC5AC** ^ +^ **, MUC2** ^ +^ **, CDX-2** ^ +^ (MUC1^−^, MUC6^−^)	**Colloidal ** carcinoma
**Pancreatobiliary ** (MD-IPMN)	**MUC5AC** ^ +^, **MUC1** ^+^ (MUC2^−^, MUC6^+/−^)	**Tubular (ductal)** carcinoma
**Gastric** (BD-IPMN)	**MUC5AC** ^ +^, **(MUC6** ^+^ **)** (MUC1^−^, MUC2^−^)	**Tubular (ductal) ** carcinoma
**Oncocytic** (MD-IPMN)	**MUC5AC** ^ +^, **MUC6** ^+^ (MUC2^+/−^) (MUC1^+/−^)	**Oncocytic ** carcinoma

**Table 2:** Core characteristics of cystic lesions of the pancreas (adapted from [47]).

	IPMN	SCN	MCN	SPN	Pseudocysts
Mean age (years)	60–70	70	40–50	30	30–50
Sex	60–70% male	90% female	>95% female	90% female	70–80% male
Localization (average)	Pancreatic head	Pancreatic tail	Pancreatic body and tail	Pancreatic body and tail	Pancreas ubiquitary
Imaging and MPD	Segmental or diffuse enlargement of the MPD and obligatory communication to the MPD	Microcystic lesion with central scar and calcification (or macrocystic lesion without central scar possible); no connection to the MPD	Macrocystic lesion with septation and calcification of the wall; no connection to the MPD	Mixed solid cystic lesion; no connection to the MPD	Macrocystic lesion without septation; connection to the MPD and probably signs of pancreatitis; enlargement of MPD possible
CEA in cyst	High	Low	High	Low	Low
Mucin production	Yes	No	Yes	No	No
Amylase in cyst	High	Low	Low	Low	High

MPD: main pancreatic duct; CEA: carcinoembryonic antigen; IPMN: intraductal papillary mucinous neoplasm; SCN: serous cystic neoplasm; MCN: mucinous cystic neoplasm; SPN: solid pseudopapillary neoplasm.

## Abbreviations

PanIN: Pancreatic intraepithelial neoplasia
PDAC: Pancreatic ductal adenocarcinoma
IPMN: Intraductal papillary mucinous neoplasm
MCN: Mucinous cystic neoplasm
MD-IPMN: Main duct intraductal papillary mucinous neoplasm
BD-IPMN: Branch duct intraductal papillary mucinous neoplasm
SPN: Solid pseudopapillary neoplasm
SCN: Serous cystic neoplasm.
